# Prostaglandin E2 produced by myeloid-derived suppressive cells induces cancer stem cells in uterine cervical cancer

**DOI:** 10.18632/oncotarget.26347

**Published:** 2018-11-20

**Authors:** Hiromasa Kuroda, Seiji Mabuchi, Eriko Yokoi, Naoko Komura, Katsumi Kozasa, Yuri Matsumoto, Mahiru Kawano, Ryoko Takahashi, Tomoyuki Sasano, Kotaro Shimura, Michiko Kodama, Kae Hashimoto, Kenjiro Sawada, Eiichi Morii, Tadashi Kimura

**Affiliations:** ^1^ Department of Obstetrics and Gynecology, Osaka University Graduate School of Medicine, Osaka, Japan; ^2^ Department of Pathology, Osaka University Graduate School of Medicine, Osaka, Japan

**Keywords:** MDSC, CSC, prostaglandin E2, cervical cancer, celecoxib

## Abstract

Myeloid-derived suppressor cells (MDSCs) enhance tumor progression by suppressing tumor-specific T cell responses, stimulating tumor angiogenesis, or promoting tumor cell metastasis. However, the biology of MDSCs have not been fully investigated. In the current study, we investigated the role of MDSCs in inducing cancer stem-like cells and explored a clinically feasible approach for targeting MDSCs-mediated cancer stem-like cells induction. *In vitro* and *in vivo* experiments revealed that MDSCs induced by tumor-derived G-CSF enhanced the stemness of cervical cancer cells by producing Prostaglandin E2 (PGE2). We also demonstrated that anti-Gr-1 neutralizing antibody or celecoxib inhibited the induction of cancer stem-like cells and enhanced the efficacy of cisplatin in cervical cancer. In clinical samples, MDSCs, PGE2, and CSCs had positive correlations. In conclusion, G-CSF-induced MDSCs enhance the stemness of uterine cervical cancer cells by producing PGE2. Targeting MDSCs or PGE2 might be a reasonable strategy for enhancing the efficacies of treatments.

## INTRODUCTION

Uterine cervical cancer is the second most common type of cancer among women worldwide, and 530,000 new cases occur globally each year [1]. Although most patients can be cured with treatments based on surgery and/or chemoradiotherapy, a significant number of patients experience treatment failures: the risk of recurrence is 10–20% for FIGO (The International Federation of Gynecology and Obstetrics) stages IB-IIA and 50–70% in stages IIB-IVA [2]. To improve the prognosis of cervical cancer patients, the biological mechanisms responsible for the treatment resistance need to be investigated.

Interactions between tumor cells and the host immune system causes immunoediting, stimulates tumor immune evasion, and ultimately result in tumor invasion, metastasis, and relapse [3]. Myeloid-derived suppressor cells (MDSC) are an important immune component in the tumor microenvironment and are considered to mediate immune suppression in tumor-bearing mice and cancer patients [4]. In addition to immune suppression, MDSCs have also been demonstrated to enhance tumor progression by stimulating cancer cell invasion, metastasis, and tumor angiogenesis [4]. However, the biology of MDSCs has not been fully investigated.

Cancer stem-like cells (CSC) are a subpopulation of tumor cells that are considered to contribute to tumor initiation, progression, metastasis, and therapeutic resistance. Experimental evidence supporting the existence of CSCs was first reported in 1997 [5]. Since then, an increasing number of investigators have identified CSCs in a variety of malignancies [6]. Considering the characteristics of CSCs, treatment failures after standard treatments could be explained, at least in part, by the CSC hypothesis. Thus, the development of treatments targeting CSCs is urgently needed. In uterine cervical cancer, previous studies have demonstrated the presence of CSCs, and identified CSCs were associated with resistance to treatments [7–10]. However, the precise mechanisms by which CSCs are induced and maintained in cervical cancer microenvironment remain unknown.

It has been demonstrated that tumor-associated macrophages (TAM) or cancer-associated fibroblasts (CAFs) enhanced the stemness in experimental model of human malignancies [11], indicating that cancer stemness is partly defined by bone marrow-derived cells. Recently, several studies have shown the possibility that that MDSCs are involved in the CSC-induction in experimental models of ovarian or pancreatic cancer [12–14]. Moreover, a recent study suggested that prostaglandin E2 (PGE2) from tumor microenvironment or inflammation enhance the stemness of colorectal cancer cells [15]. However, due to the small number of published reports, whether MDSCs stimulate the stemness of cancer cells and the underlying mechanism responsible for MDSC-mediated CSC-regulation remain to be elucidated.

We have recently demonstrated that tumor-derived granulocyte-colony stimulating factor (G-CSF) stimulates cervical cancer progression through the induction of MDSCs [16, 17]. We also showed that MDSCs induced by tumor-derived G-CSF were involved in the resistance of cervical cancer to radiotherapy [16] and platinum-based chemotherapy [17]. These results indicate that there is a possibility that MDSCs induce CSCs in cervical cancer microenvironment, especially those expressing G-CSF.

Previous studies have suggested that tumor-derived PGE2 induces and activates MDSCs [18]. MDSCs, on the other hands, express high levels of cyclooxygenase-2 (COX2) and produces PGE2 [19]. A recent report indicates that MDSC-derived PGE2 is involved in the suppressive activity of MDSCs on CD8-positive T cells [20]. Collectively, these results strongly indicate the significance of the positive feedback between COX-2 and PGE2 in MDSC [19]. However, the associations between G-CSF, MDSCs, PGE2 and CSCs have never been investigated.

In the current study, using clinical samples, cervical cancer cell lines, and a mouse model of cervical cancer, we investigated the role of MDSCs in the induction of CSCs in uterine cervical cancer. We also investigated practical clinical methods for inhibiting MDSC-mediated CSC-induction and enhancing the efficacy of existing treatments.

## RESULTS

### The self-renewal, tumorigenic, and differentiating capacities of ALDH-high ME180 cells

As high aldehyde dehydrogenase (ALDH) activity was reported to be a marker of CSCs in various cancers [21, 22], we first investigated the ALDH activity of 2 cervical cancer cell lines (ME180 and CaSki). As shown in Supplementary Figure 1, each of the 2 cervical cancer cell lines contained ALDH-high cells. ALDH activity was detected in 1.6% and 3.7% of the ME180 and the CaSki cells, respectively.

**Figure 1:**
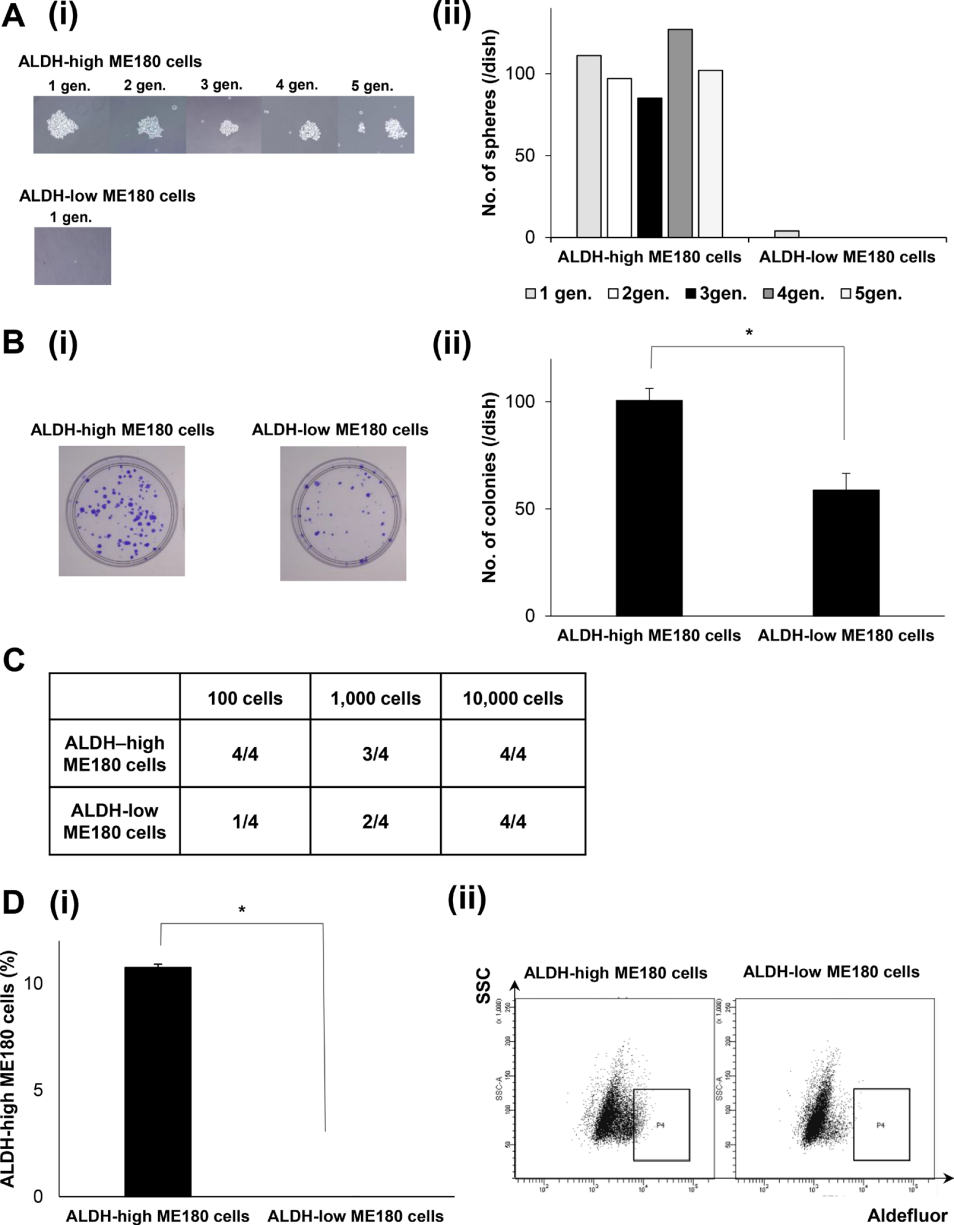
Cancer stem-like properties of ALDH-high ME180 cells (**A**) *In vitro* tumorigenic capacity and self-renewal activity of cervical cancer cells according to ALDH-activity. ME180 cells that had been labeled with the Aldefluor kit were sorted using a FACScan flow cytometer. Then, ALDH-high and ALDH-low ME180 cells (1.5 × 10^3^ cells) were separately plated in 60 mm ultra-low attachment surface dishes and cultured for 2 weeks in the serum-free medium. The tumorigenic capacity and self-renewal activity of the cells were assessed by sphere formation assays (i), Representative photos of the spheres formed by the ALDH-high and ALDH-low cells (phase-contrast microscopy, magnification: ×400). (ii) The number of spheres counted from 5 consecutive passages (*n* = 3, *p* < 0.05, two-sided Student’s *t* test). (**B**) *In vitro* tumorigenic capacity of cervical cancer cells according to ALDH-activity. ME180-high and ME180-low cells (1 × 10^2^) were separately cultured in 60 mm dishes in the presence of 10% FBS for 3 weeks. Then, the colonies were stained with 0.5% crystal violet and the numbers of colonies were counted. (i) Representative photos of colonies. (ii), The numbers of colonies counted (Bars SD, *n* = 3. *p* < 0.05, two-sided Student’s *t* test). (**C**) *In vivo* tumorigenic capacity of cervical cancer cells according to ALDH-activity. ALDH-high or low ME180 cells were subcutaneously inoculated into NOD/SCID mice (100 cells; 1,000 cells; 10,000 cells). Eight weeks after the inoculation procedure, the numbers of successful tumor initiations for each condition were counted and shown (*n* = 4). (**D**) Differentiation capacity of cervical cancer cells according to ALDH-activity. (i) Population of ALDH-high cells. ALDH-high and ALDH-low ME180 cells were separately cultured in the presence of 10% FBS for 3 days *in vtiro* and then assessed using the Aldefluor assay (Bars SD, *n* = 5, *p* < 0.01, two-sided Student’s *t* test). (ii) Representative dot plots were shown.

The stem cell-like property of ALDH-high CaSKi cells have been demonstrated in a previous study [21, 23]. Thus, Using ME180 cells, we next investigated the stemness of ALDH-high ME180 cells. To assess their tumorigenicity and self-renewal capacity *in vitro*, ALDH-high and ALDH-low ME180 cells were cultured in ultra-low attachment surface dishes under serum-free conditions. As shown in Figure 1A, the ALDH-high ME180 cells generated tumor-spheres in 5 consecutive passages. In contrast, the ALDH-low ME180 cells did not form tumor-spheres.

To determine the tumorigenic capacity of ALDH-high ME180 cells *in vitro*, we next performed *in vitro* colony formation assays. As shown in Figure 1B, the ALDH-high ME180 cells formed greater numbers of colonies than the ALDH-low ME180 cell. The tumorigenic capacity of ALDH-high ME180 cells was also confirmed in an *in vivo* experimental model. Limited numbers of ALDH-high ME180 or ALDH-low ME180 cells were subcutaneously inoculated into NOD/SCID mice. As shown in Figure 1C, all NOD/SCID mice inoculated with 10^2^ ALDH-high ME180 cells successfully formed subcutaneous tumors. In contrast, only 1 out of the 4 NOD/SCID mice inoculated with 10^2^ ALDH-low ME180 cells developed a subcutaneous tumor. These results from *in vitro* and *in vivo* experiments suggest that ALDH-high ME180 cells have tumorigenic capacity.

To assess their differentiation potential, ALDH-high and ALDH-low ME180 cells were separately cultured for 3 days, and then the ALDH activities of the cultured populations were analyzed using the Aldefluor assay. As shown in Figure 1D, approximately 90% of the ALDH-high ME180 cells differentiated into ALDH-low cells, and 10% of the cells remained strongly ALDH-high. In contrast, more than 99% of the ALDH-low ME180 cells retained the ALDH-low phenotype. Collectively, these results suggest that ALDH-high ME180 cells are highly tumorigenic and have self-renewal and differentiation capacities.

### The radio- or chemoresistant nature of the ALDH-high ME180 cells

**Figure 2:**
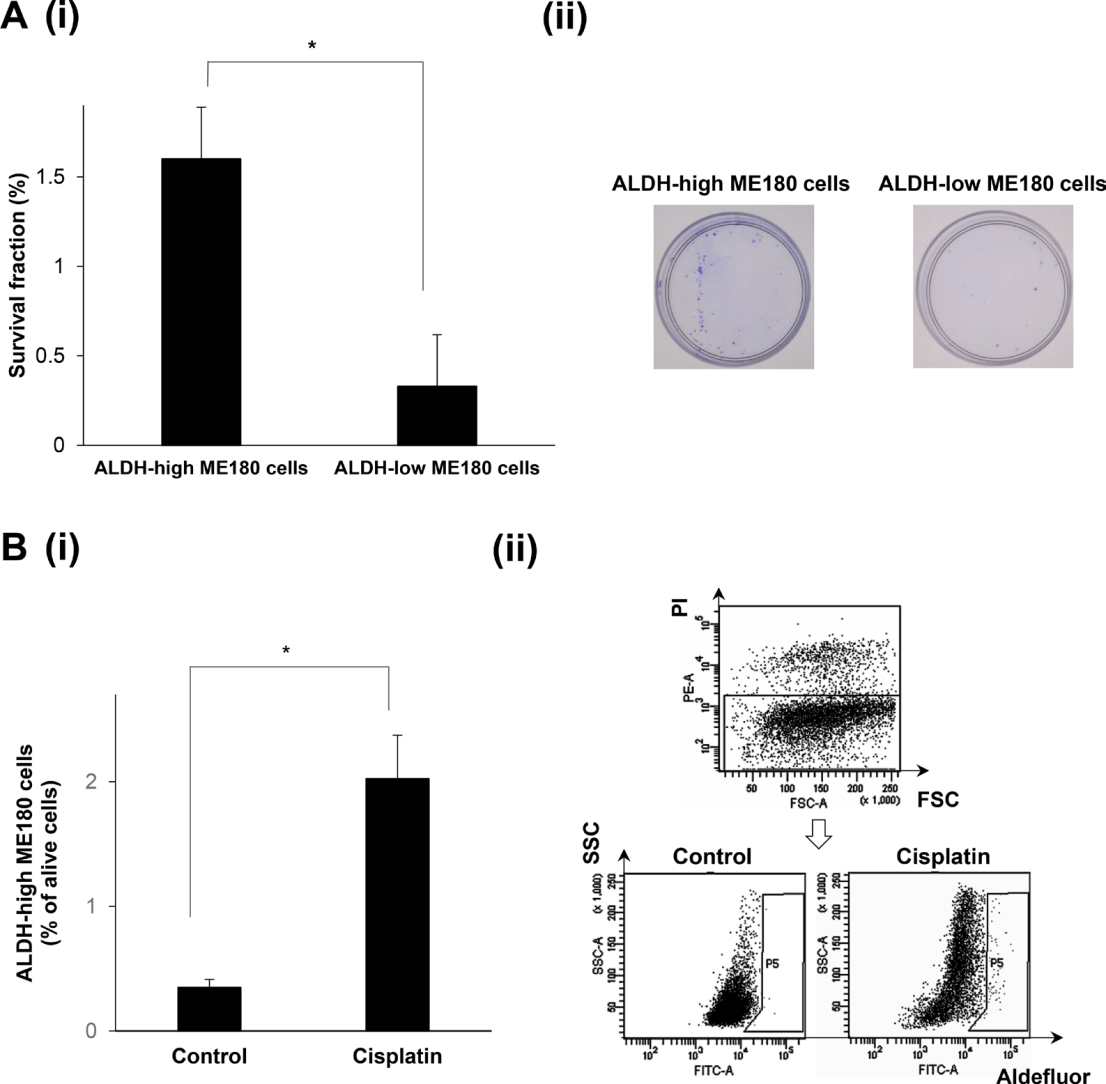
The radio- or chemoresistant nature of the ALDH-high ME180 cells (**A**) Radioresistant nature of the ALDH-high ME180 cells assessed by clonogenic survival assay. ALDH-high and ALDH-low ME180 cells (1 × 10^3^) were severally plated in 60 mm dishes and treated with 4 Gy of radiotherapy in the presence of 10% of FBS. As a control group, 100 cells of ALDH-high and ALDH-low ME180 cells were plated. After they had been cultured for 3 weeks, the colonies were stained with 0.5% crystal violet and the numbers of colonies were counted. The survival fractions (SF) were calculated by following formulas. SF = 100 × plating efficacy (PE) of treated sample/PE of control. PE = number of colonies/number of cells plated. (i) The survival fractions of ALDH-high and ALDH-low ME180 cells (Bars SD. *n* = 3, *p* < 0.01, two-sided Student’s *t* test). (ii) Representative photos of the colonies formed by the ALDH-high and ALDH-low cells treated with 4 Gy of radiotherapy. (**B**) Chemoresistant nature of the ALDH-high ME180 cells. ME180 cells (3 × 10^6^) were inoculated with 1 mM of cisplatin or PBS (control) in the presence of 10% of FBS for 3 days in 100 mm dishes. Among the surviving ME180 cells (propidium iodide (PI)-negative cells), the percentages of ALDH-high cells were assessed using the Aldefluor assay. (i) The percentage of ALDH-high ME180 cells (Bars SD. *n* = 4, *p* < 0.05, two-sided Student’s *t* test). (ii) Representative dot plots are shown.

To assess the radioresistant nature of ALDH-high cells, we next performed clonogenic survival assays (Figure 2A). As shown, significantly greater numbers of colonies were formed by the ALDH-high ME180 cells than by the ALDH-low ME180 cells after the treatment with 4 Gy of radiotherapy. We next investigated the chemoresistant natures of ALDH-high ME180 cells. For this purpose, we employed cisplatin, a key cytotoxic agent in the treatment of cervical cancer. As shown in Figure 2B, in the cisplatin-untreated condition, ALDH-high cells accounted for 0.3% of ME180 cells. In contrast, when ME180 cells were treated with 1μM cisplatin for 3 days, ALDH-high cells were detected at a frequency of 2%, which indicating the cisplatin-resistant nature of ALDH-high ME180 cells. Overall, these results indicate that ALDH-high ME180 cells are resistant to chemotherapy and radiotherapy.

### The correlation between MDSCs and ALDH-high tumor cells

**Figure 3:**
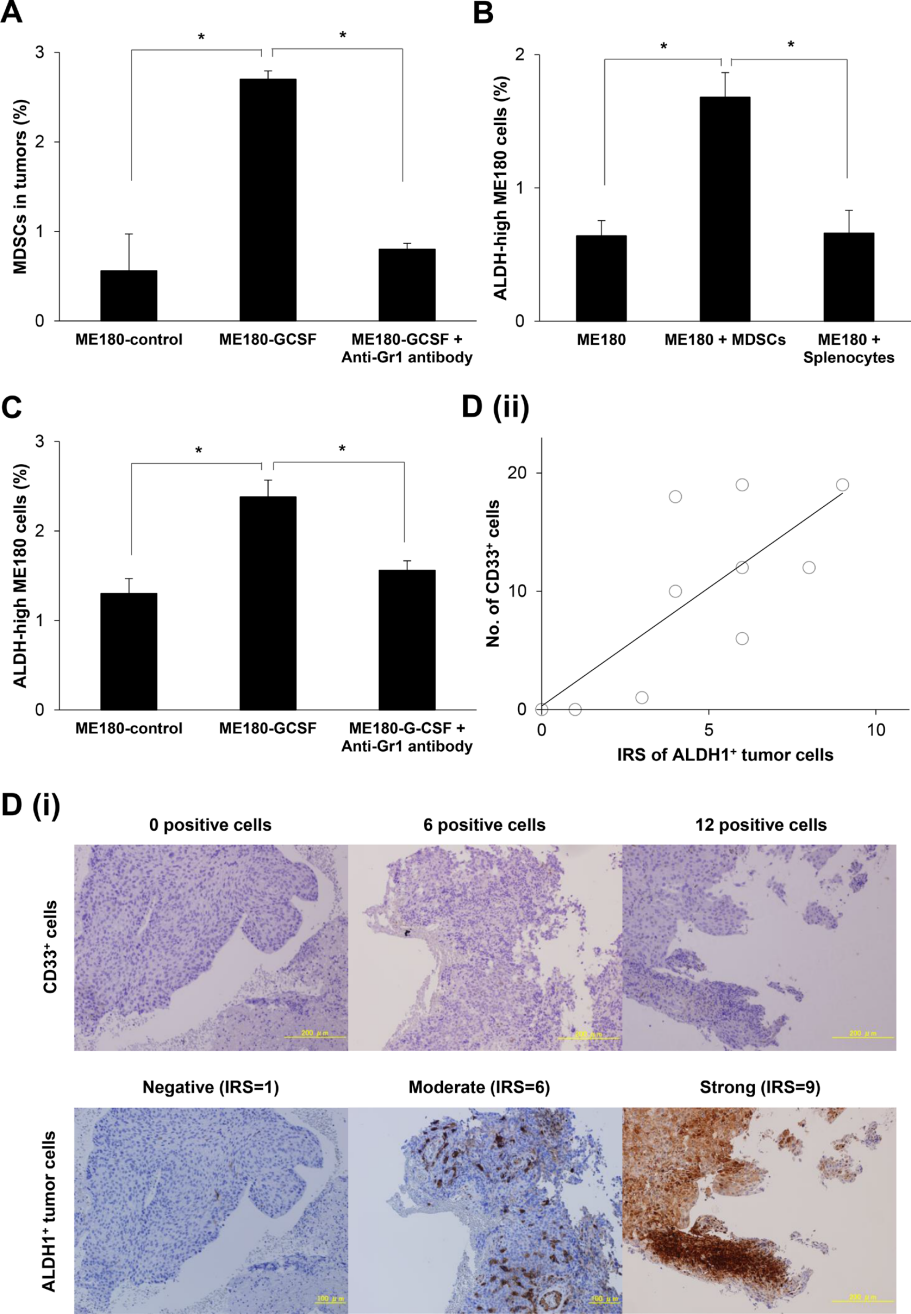
The correlation between MDSCs and CSCs (**A**) Induction of MDSCs by tumor-derived G-CSF. Balb/c mice were inoculated with ME180-GCSF (*n* = 10) or ME180-control cells (*n* = 5). Five of the mice bearing ME180-GCSF-derived tumors were treated with anti-Gr-1 antibody at a dose of 200 μg/mouse. Four weeks after the inoculation, their subcutaneous tumors were collected and assayed for MDSCs using flow cytometry (Bars SD. *n* = 5, *p* < 0.01, two-sided Student’s *t* test). (**B**) Effect of MDSCs in the induction of CSCs *in vitro*. ME180 cells were cultured with or without MDSCs in the presence of 0.1% of FBS for 12 hours (5:1 ratio of ME180: MDSCs). The frequencies of ALDH-high ME180 cells were assessed using the Aldefluor assay. Splenocytes (excluding MDSCs) were used as negative control. (Bars SD. *n* = 6, *p* < 0.01, two-sided Student’s *t* test). (**C**) The frequency of CSCs in an *in vivo* cervical cancer model. Balb/c nude mice were subcutaneously inoculated with ME180-GCSF (*n* = 10) or ME180-control cells (*n* = 5). Five of the mice bearing ME180-GCSF-derived tumors were treated with anti-Gr-1 antibody at a dose of 200 μg/mouse. Four weeks after the inoculation, their subcutaneous tumors were collected. The human EpCam^+^ mouse CD45^-^ cells in the tumors were gated by flow cytometry, and then the percentages of ALDH-high cells were assessed using the Aldefluor assay (Bars SD. *n* = 5, *p* < 0.01, two-sided Student’s *t* test). (**D**) The association between tumor-infiltrating MDSCs and CSCs in human cervical cancer. Cervical cancer biopsy samples that were obtained at the initial diagnosis (*n* = 10) were stained with anti-CD33 or anti-ALDH1 antibodies. The number of CD33^+^ cells was counted using a blight field microscope in low-power fields. The ALDH1 immunoreactivity of tumor cells was assessed using an immunoreactive score according to Remmele and Stegner (IRS). (i) Representative photographs. Magnification: × 80. (ii) Correlation between the number of CD33^+^ cells and ALDH-high ME180 cells. A positive correlation was detected between the CD33^+^ cells and ALDH-high cells (Spearman’s correlation coefficient, *r* = 0.741, *p* < 005).

We next investigated whether MDSCs enhance ALDH-activity of cervical cancer cells *in vitro*. To obtain MDSCs, we inoculated Balb/c nude mice with ME180 cells that had been stably transfected with G-CSF (ME180-GCSF). ME180 cells stably transfected with control vector (ME180-control) was also established and used as a control. As shown in Figure 3A and Supplementary Figure 2A, the mice bearing ME180-GCSF-derived tumors displayed significantly greater numbers of MDSCs (CD11b^+^Gr-1^+^) in their subcutaneous tumors than the mice bearing ME180-control-derived tumors.

We then investigated the subset of MDSCs obtained from ME180-GCSF-derived tumors using flow cytometry. As shown in Supplementary Figure 2B, granulocytic MDSCs (CD11b^+^Ly-6G^+^Ly-6C^low^cells) accounted for more than 80% of CD11b cells, whereas monocytic MDSCs (CD11b^+^Ly-6G^-^Ly-6C^+^cells) less than 10%, indicating that these granulocytic MDSC were the dominant subset that was expanded by the tumor-derived G-CSF in this experimental model.

Using MDSCs that had been extracted from the spleens of mice bearing ME180-GCSF-derived tumors, we conducted co-culture experiments. As shown, when ME180 cells were co-cultured with the MDSCs, the frequency of ALDH-high ME180 cells was significantly increased (Figure 3B), which was in clear contrast with result obtained when ME180 cells were co-cultured with splenocytes. Similar results were seen when CaSki cells were co-cultured with MDSCs (Supplementary Figure 2C).

We then investigated whether MDSCs enhanced the stemness of cervical cancer cells *in vivo*. As shown in Figure 3C and Supplementary Figure 2D, significantly increased numbers of ALDH-high ME180 cells were observed in the ME180-GCSF-derived tumors that contained increased numbers of MDSCs than in ME180-control-derived tumors. On the basis of previous reports showing the ability of anti-Gr-1 antibody (RB6-8C5) to eliminate MDSC *in vivo* [16, 24, 25], we next investigated the efficacy of anti-Gr-1 antibody in our experimental models. As shown, when the mice bearing ME180-GCSF-derived tumors were treated with anti-Gr- 1 antibody, the frequencies of MDSCs and ALDH-high ME180 cells in tumor fell significantly to similar levels to those seen in the ME180-control-derived tumors (Figure 3A and 3C; Supplementary Figure 2A and 2D).

To confirm the association between MDSCs and ALDH-high tumor cells in human cervical cancer specimens, we conducted immunohistochemical analyses of CD33 and ALDH1 expression using biopsy samples that were obtained at the initial diagnosis. As shown in Figure 3D (i), cervical cancer specimens exhibited various degrees of immunoreactivities for CD33 (upper panel) and ALDH1 (lower panel). Of the 10 examined cases, 1 (10%), 6 (60%), 1 (10%), and 2 (20%) were categorized as strong (IRS = 9–12), moderate (IRS = 4–8), weak (IRS = 2–3), and negative (IRS = 0–1), respectively. Moreover, as shown in Figure 3D (ii), positive correlation was observed between the number of CD33 positive cells and IRS scores of ALDH1 expression (Spearman’s correlation coefficient, *r* = 0.741, *P* < 005).

### The mechanism responsible for MDSC-mediated enhancement of stemness in cervical cancer

**Figure 4:**
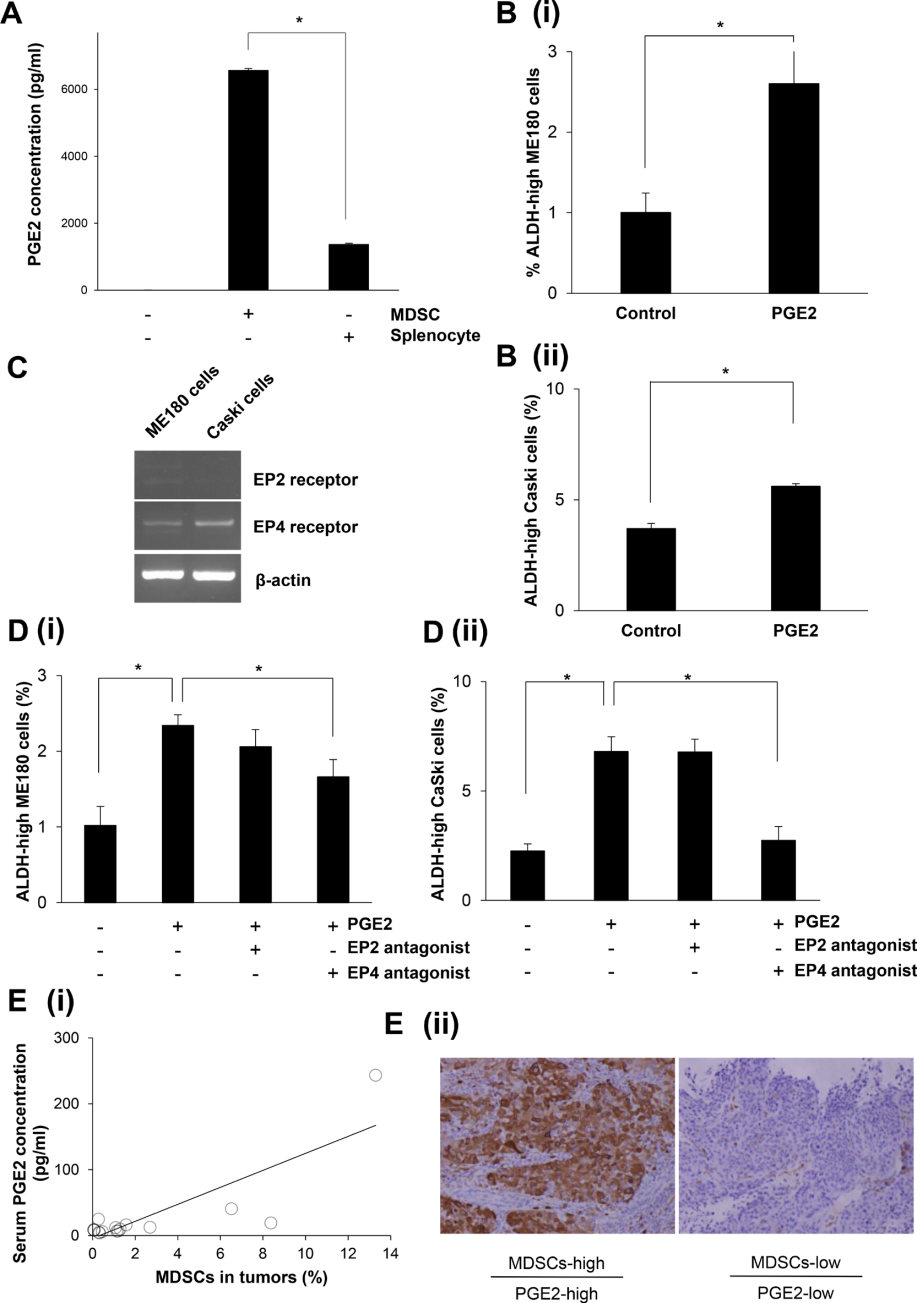
The mechanism responsible for the enhancement of stemness by MDSCs (**A**) Production of PGE2 by MDSCs. MDSCs that had been isolated from spleens of mice bearing ME180-GCSF-derived tumors were cultured in serum-free medium. Splenocytes (excluding MDSCs) were also used as a comparison. The PGE2 concentrations of the culture media were measured by the Prostaglandin E2 Express ELISA Kit. (Bars SD. *n* = 3, *p* < 0.01, two-sided Student’s *t* test). (**B**) Effect of PGE2 on the induction of cervical cancer CSCs *in vitro*. ME180 (i) or CaSki (ii) cervical cancer cells were treated with 0.1 μM of PGE2 in the absence of FBS for 18 hours *in vitro*. Then, the frequencies of ALDH-high ME180 cells were assessed using the Aldefluor assay. (Bars SD. *n* = 6, *p* < 0.01, two-sided Student’s *t* test). (**C**) Expressions of the EP2 or EP4 receptor in cervical cancer cells. The EP2 receptor, the EP4 receptor, and β-actin mRNA levels of ME180 cells and CaSki cells that had been incubated in the presence of 10% of FBS were assessed by RT-PCR. (**D**) Effect of EP2/EP4-inhibition on the PGE2-mediated CSC-induction. ME180 (i) or CaSki (ii) cervical cancer cells were treated either with 0.1 μM of PGE2, an EP2 antagonist, or an EP4 antagonist in the absence of FBS for 18 hours *in vitro*. Then, the frequencies of ALDH-high tumor cells were assessed using the Aldefluor assay. (Bars SD. *n* = 6, *p* < 0.05, two-sided Student’s *t* test). (**E**) (i) Correlation between the number of MDSCs (CD11b^+^CD33^+^HLA-DR^-^ cells) and the serum PGE2 concentration in cervical cancer patients. Using biopsy samples that were obtained from cervical cancer patients at the initial diagnosis, CD11b^+^CD33^+^HLA-DR^-^ cells were counted using flow cytometry. Blood samples were obtained from the same patients. Their serum PGE2 concentrations were measured using the Prostaglandin E Metabolite ELISA Kit. A positive correlation was detected (Spearman’s correlation coefficient, *r* = 0.×, *p* < 005). (ii) ALDH1 immunoreactivity in cervical cancer specimens. Cervical cancers obtained from patients exhibiting increased numbers of tumor MDSCs and elevated serum PGE2 concentrations (upper panel), and from those exhibiting decreased numbers of tumor MDSCs and low serum PGE2 concentrations (lower panel) were stained with anti-ALDH1 antibodies. Representative images of primary tumors are shown. (magnification: ×200).

To investigate the mechanism by which MDSCs enhance the stemness of cervical cancer, we first examined whether MDSCs produce PGE2 *in vitro* by ELISA, as it has been recently reported that PGE2 enhances the stemness of colorectal or bladder cancer cells [15, 26]. As shown in Figure 4A, the MDSCs extracted from the spleens of the mice bearing ME180-GCSF-derived tumors produced PGE2, which was in clear contrast to the splenocytes (excluding MDSCs) extracted from the same mice.

To investigate the role played by MDSC-derived PGE2 in the enhancement of the stemness of uterine cervical cancer cells, ME180 cells and CaSki cells were treated with the indicated concentration of PGE2 *in vitro*. The numbers of ALDH-high ME180 cells and ALDH-high CaSki cells were significantly increased in response to the treatment with PGE2 (Figure 4B; Supplementary Figure 3A and 3B). We then investigated the expression status of the EP2 and EP4 receptors, which are known to be involved in PGE2-signaling, in ME180 and CaSki cells. As shown in Figure 4C, both the ME180 cells and CaSki cells expressed the EP4 receptor. However, the EP2 receptor was not expressed in these cell lines. Consistent with these findings regarding prostanoid receptor expression, the PGE2-mediated induction of ALDH-high cells was significantly inhibited by treatment with an EP4 antagonist (ONO-AE3-208) *in vitro* (Figure 4D). Collectively, these results indicate that the PGE2 produced by MDSCs enhances the stemness of cervical cancer cells.

To determine whether the findings obtained in mice are representative of the clinical status of cervical cancer patients, we investigated the numbers of MDSCs in the blood and the serum PGE2 concentrations in patients with newly diagnosed cervical cancer. For this purpose, we employed CD11b^+^CD33^+^HLA-DR^-^ to define human MDSCs according to the method described in a previous study [Supplementary Figure 3C] [16]. Consistent with the findings obtained in mice, the serum PGE2 concentrations of the patients whose cervical tumors contained higher numbers of MDSCs were significantly greater than those of the patients whose cervical tumors contained lower numbers of MDSCs (Figure 4E). Furthermore, as shown in Figure 4E (ii), in the tumors obtained from patients exhibiting increased numbers of tumor MDSCs and elevated serum PGE2 concentrations, strong ALDH1 expression of the tumor cells was observed. Overall, these results strongly suggest that the MDSC-PGE2-CSC axis demonstrated in our mouse model of cervical cancer is applicable to human cervical cancer.

### Effects of celecoxib on the MDSC-mediated enhancement of stemness in cervical cancer

**Figure 5:**
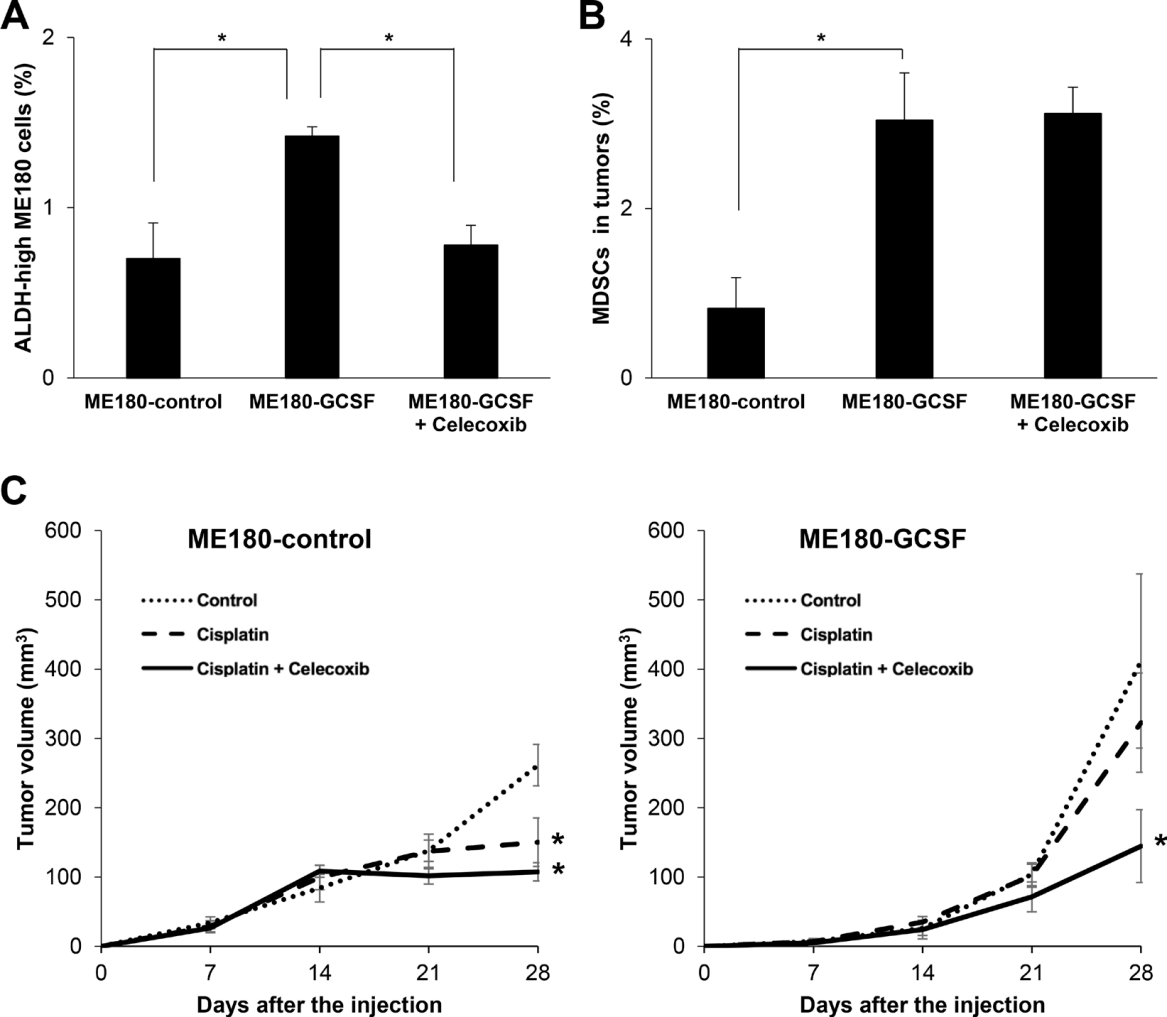
Effects of PGE2-inhibition using with celecoxib (**A**, **B**) *In vivo* effect of PGE2-inhibition on the induction of CSCs. Balb/c nude mice were inoculated with ME180-GCSF (*n* = 10) or ME180-control cells (*n* = 5). Two weeks after the inoculation, mice bearing ME180-GCSF-derived tumors were randomly assigned to 2 treatment groups: 5 mg/kg of daily celecoxib (i.p.) or PBS. Four weeks after the start of the treatment, their subcutaneous tumors were collected for evaluation. (A) Effect of celecoxib on the induction of CSCs in tumor. The human EpCam^+^ mouse CD45^-^ cells in the tumors were gated using flow cytometry and then the percentages of ALDH-high cells were assessed using the Aldefluor assay (Bars SD. *n* = 5, *p* < 0.01, two-sided Student’s *t* test). (B) Effect of celecoxib on the induction of MDSCs in tumor. The percentages of MDSCs (CD11b^+^Gr-1^+^) in the tumors were assessed using flow cytometry (Bars SD. *n* = 5, *p* < 0.01, two-sided Student’s *t* test). (**C**) Anti-tumor effect of the celecoxib-mediated inhibition of PGE2-CSC axis in a mouse cervical cancer model. Balb/c nude mice were inoculated with ME180-GCSF or ME180-control cells. Two weeks after the inoculation, the mice were assigned to 4 treatment groups: PBS (*n* = 5), 5 mg/kg of daily celecoxib (*n* = 5), 4 mg/kg of weekly cisplatin (*n* = 5), or 4 mg/kg of weekly cisplatin combined with 5 mg/kg of daily celecoxib (*n* = 5). The volumes of the tumors were measured for 4 weeks after the inoculation (Bars SD. *n* = 5, *p* < 0.05, two-sided Student’s *t* test).

To further demonstrate the involvement of PGE2, we investigated the effects of celecoxib on the induction of ALDH-high tumor cells *in vivo*. According to previous studies, the sulfonamide side chain of celecoxib binds to a hydrophilic side pocket region close to the active COX-2 binding site, resulting in the selective inhibition of the COX-2 activity and PGE2 [27]. We first examined the COX-2 expression in MDSC. As shown (Supplementary Figure 4A), consistent with the previous reports showing that COX-2 is the dominant subset in white blood cells or spleen [28], the expression of COX-2 is greater than COX-1. We next confirmed that, when mice bearing ME180-GCSF-derived tumors were treated with celecoxib, the serum PGE2 concentrations of the mice were significantly decreased (Supplementary Figure 4B). Moreover, treating mice bearing ME180-GCSF-derived tumors with celecoxib significantly reduced the frequency of ALDH-high ME180 cells in the tumors to the same level as was seen in the ME180-control- derived tumors. However, the celecoxib treatment did not result in the decrease in MDSC frequency (Figure 5A and 5B; Supplementary Figure 4C) or the reduction in the size of ME180-GCSF-derived tumors (Supplementary Figure 4D).

Finally, we investigated whether the inhibition of MDSC-mediated induction of ALDH-high tumor cells by celecoxib might enhance the anti-tumor efficacy of cisplatin *in vivo*. As shown in Figure 5C, although cisplatin significantly inhibited the growth of ME180-control-derived tumors, the growth-inhibitory effect of cisplatin was minimal against the ME180-GCSF-derived tumor, which indicated the relatively cisplatin-resistant nature of ME180-GCSF-derived tumor. When celecoxib was administrated in combination with cisplatin, celecoxib significantly sensitized the ME180-GCSF-derived tumors to cisplatin. However, the effect of celecoxib was minimal in the mice bearing ME180-control-derived tumors. These results indicate that the inhibition of PGE2 production by celecoxib depressed CSC-maintenance, which resulted in an increase in the anti-tumor efficacy of cisplatin.

## DISCUSSION

In the current study, we have shown that G-CSF-induced MDSCs enhanced the stemness of cervical cancer cells by producing PGE2. We also demonstrated that the inhibition of MDSCs or PGE2 inhibited the induction of CSCs effectively and enhanced the efficacy of cisplatin in experimental models of cervical cancer. Moreover, in the analyses using patients-derived samples, we have confirmed that the frequency of tumor-infiltrating MDSCs is positively correlated with the frequency of CSCs in tumor as well as the serum PGE2 concentration in cervical cancer patients. These results indicate that MDSC-PGE2-CSC axis demonstrated in our *in vitro* or *in vivo* experiments is applicable to human cervical cancer.

The involvement of MDSCs in the induction of CSCs has been shown in several studies. The first study was conducted by Cui TX *et al*, and showed that MDSCs enhanced the stemness of ovarian cancer cells [12]. They demonstrated that MDSCs triggered miRNA-101 expression in ovarian cancer cells, and miRNA-101 subsequently repressed the corepressor gene C-terminal binding protein-2 (CtBP2), resulting in enhanced cancer cell stemness. In their study, neither the mechanism by which MDSCs triggers miRNA-101 expression in cancer cells nor the subpopulation of MDSCs (i.e., granulocytic or monocytic) was not investigated. Subsequently, Panni RZ *et al.* reported that monocytic MDSCs enhanced the stemness of pancreatic cancer cells via the production of interleukin (IL)-6 and the subsequent activation of signal transducer and activator of transcription 3 (STAT3) activation in cancer cells [13]. Peng D *et al.* have recently shown that MDSCs enhance the stemness of breast cancer cells through the production of IL-6 and nitric oxide (NO) and the subsequent activation of STAT3 and Notch signaling pathways, respectively. However, the subpopulation of MDSCs was not investigated in their study [14]. To the best of our knowledge, the current study is the first to demonstrate the involvement of MDSCs in the induction of CSCs in cervical cancer. Moreover, we showed, for the first time, that granulocytic MDSCs contributed in the induction of ALDH-high tumor cells (Figure 3A–3C; Supplementary Figure 2A and 2C).

Recently, two previous studies showed that PGE2 enhances the stemness of cancer cells. One was an investigation in colorectal cancer, and the other was a study of bladder cancer [15, 26]. In the both studies, PGE2 produced by cancer cells promoted the induction of CSCs via the autocrine pathway. Thus, the current study discovered a new mechanism responsible for the PGE2-mediated induction of CSCs: i.e., MDSC-derived PGE2 is involved in the induction of CSCs.

The findings obtained in the current study could have important clinical implications. By assessing the pretreatment MDSC frequency or the PGE2 concentration in the peripheral blood, it might be possible to identify high-risk cervical cancer patients who would have high probability to show resistance to conventional standard treatments including chemotherapy or radiotherapy. We consider that the combination of conventional treatments (i.e., chemotherapy or radiotherapy) with CSC-targeting therapies (i.e., MDSCs- or PGE2-targeting therapy) may be effective in these patients. Currently, no specific inhibitor of human MDSCs has yet been identified. Previous study has shown that selective COX-2 inhibitor celecoxib reduces the numbers and the suppressive functions of MDSC [20, 28]. Treatment MDSC with celecoxib also inhibited the production of PGE2 via the selective inhibition of COX-2 [28]. Moreover, other studies have shown that inhibition of tumor-derived PGE2 either by a COX-2 gene silencing [18] or a COX-2 inhibitor celecoxib [29] effectively suppressed the tumor growth by Inhibiting MDSC. Based on these rationales, we have employed celecoxib in the current study. Celecoxib has demonstrated its safety and the efficacy as an antiulcer drug or a painkiller in the systematic review [30] and is now widely used in clinical practice. Thus, we consider that PGE2-targeting therapy using celecoxib might be the most clinically practical option. In a randomized phase II clinical trial of celecoxib as a treatment for cervical dysplasia, celecoxib displayed acceptable toxicity and significant clinical activity in patients with high-grade cervical dysplasia: 75% of celecoxib-treated patients achieved a clinical response, which was significantly higher than the 31% observed in the placebo-treated patients (*p* < 0.03) [31]. This result strongly indicates that PGE2 plays an integral role in the progression of cervical dysplasia to cervical cancer and supports our idea. Therefore, celecoxib might be clinically useful as a CSC-targeting agent in the treatment of cervical cancer.

For appropriate patient selection in the future clinical trials, identification of predictive biomarkers is essential. We have recently shown that MDSC-inhibition therapy was effective only against cervical cancers that contained increased numbers of MDSCs [16, 17]. In the current study, we also found that PGE2-targeting therapy was effective only against G-CSF-expressing cervical cancer that contained increased numbers of MDSCs [16, 17]. Importantly, we have demonstrated that the frequency of MDSCs in tumors can be predicted using complete blood cell counts: MDSCs are significantly increased in cervical cancer patients who display tumor-related leukocytosis (TRL). As TRL-positive cervical cancer is known to be resistant to standard chemotherapy [16, 17] and definitive radiotherapy [16], we consider that the efficacy of combining CSC-targeting therapies with conventional treatments is worth investigating in future clinical trials involving TRL-positive cervical cancer patients.

The limitations of our study need to be addressed. First, in the present study, we employed ALDH-activity as a marker of CSC, although CSC markers of any solid tumors have not been established yet. However, we confirmed the stemness of the ALDH-high cervical cancer cells in various aspects (Figure 1), several other markers have been utilized to identify and investigate human CSCs [32, 33]. Second, although we showed that non-CSCs do not dedifferentiate into CSC and that CSC differentiate into both CSC and non-CSCs (Figure 1D), a recent report indicated the possibility that non-CSCs dedifferentiate into CSCs [34]. Thus, we cannot make a definitive conclusion regarding whether non-CSC (ALDH-low cells) differentiated into CSC (ALDH-high cells) or CSC proliferated to make more CSC in response to PGE2 using the data obtained from the current study. Third, although the current study focused on the “MDSC-PGE2-CSC axis”, MDSCs are known to produce various mediators, including cytokines, chemokines, and growth factors. Thus, we have to recognize that our data do not exclude the possibility that the other MDSC-derived factors might also play roles in the induction of CSCs. Accordingly, the mechanism by which MDSCs enhances the stemness of cervical cancer cells should be investigated further. Forth, we employed nude mice in the current study, as the inoculation of human uterine cancer cells into immunocompetent mice did not result in the development of primary/metastatic tumors. Fifth, although CD33 was employed for the identification of human MDSC in the current study based on the previous studies [12, 14, 35], CD33-positive cells are not always MDSCs. Thus, clear phenotypic characterization of human MDSC by immunohistochemistry need to be investigated in the future studies. Lastly, most experiments in the current study were conducted using mouse MDSCs. Considering the future clinical development, the findings of the present study should be verified in the studies using human MDSCs.

In conclusion, we demonstrated, for the first time, that G-CSF-induced MDSCs enhanced the stemness of uterine cervical cancer cells by producing PGE2. Targeting MDSCs or PGE2 may be a reasonable strategy for enhancing the efficacies of conventional treatments including chemotherapy or radiotherapy.

## MATERIALS AND METHODS

### Patients and clinical samples

Pretreatment cervical tumor tissue and blood samples were also collected and archived according to protocols approved by the IRB of Osaka University Hospital (IRB No; 12381). Appropriate informed consent was obtained from each patient. All experiments were performed in accordance with guidelines and regulations approved by the IRB of Osaka University Hospital.

### Reagents and antibodies

The following fluorochrome-labeled antibodies were used for the staining experiments: anti-human/mouse antibodies: fluorescein isothiocyanate (FITC)-conjugated anti-CD11b (Tonbo Biosciences, San Diego, CA, USA); anti-mouse antibodies: allophycocyanin (APC)-conjugated anti-Ly6G, and phycoerythrin (PE)-conjugated anti-Ly6C (Tonbo Biosciences, San Diego, CA, USA). A neutralizing antibody against Gr-1 (RB6-8C5) was purchased from BioXCell (West Lebanon, NH, USA). The anti-tumor activity of this anti-Gr1 antibody is mediated by the antibody opsonization and antibody-dependent phagocytosis of tumor cells by macrophages [36]. Thus, we used it to eliminate MDSC only in mice studies. PGE2 was obtained from Cayman Chemical (Ann Arbor, MI, USA). Celecoxib was acquired from Sigma-Aldrich (St Louis, MO, USA). PF-04418948 (a PGE2 receptor (EP2 receptor) antagonist and ONO-AE3-208 (an EP4 receptor antagonist) were obtained from Cayman Chemical (Ann Arbor, MI, USA). Cisplatin was purchased from Sigma-Aldrich (St Louis, MO, USA).

### Drug preparation

For the *in vivo* analyses, Celecoxib was dissolved in 100% ethanol to a concentration of 20 mg/ml. Cisplatin was diluted to the appropriate concentration in double-distilled water just before its intraperitoneal infusion.

### Cell culture

MDSCs were maintained in Roswell Park Memorial Institute (RPMI)-1640 (Nacalai Tesque, Kyoto, Japan) medium supplemented with 10% fetal bovine serum (FBS). ME180 cervical cancer cells and CaSki cervical cancer cells were maintained in Dulbecco’s modified Eagle’s medium (DMEM) supplemented with 10% fetal bovine serum (FBS).

### Cell line and clone selection

ME180 cervical cancer cells were purchased from the American Type Culture Collection and passaged in our laboratory soon after they were received. ME180 cervical cancer cells were stably transfected with the G-CSF expression vector (ME180-GCSF). The expression vector for the mouse G-CSF gene (pCAmG-CSF) and the empty vector (pCAZ 2) were provided by the RIKEN BRC through the National Bio-Resource Project of the MEXT, Japan. The expression of these genes was driven by the CAG promoter, as reported previously [37, 38]. Transfection was performed using Lipofectamine 2000 (Invitrogen, Carlsbad, CA, USA), according to the manufacturer’s instructions. Clonal selection was conducted by adding G-418 to the medium at a final concentration of 500 μg/ml.

### Animal experiments

All procedures involving animals were approved by the animal care and usage committee of Osaka University, in accordance with the relevant institutional and National Institutes of Health guidelines (Approved No; 26-072-010). We employed Balb/c nude mice bearing G-CSF expressing cancer cells-derived tumors, as tumor-derived G-CSF increase the number of MDSC in mice and thus significant number of MDSC can be obtained for the experimental use [16].

Briefly, 5- to 7-week-old Balb/c mice were subcutaneously inoculated with 5 × 10^6^ of ME180-control or ME180-GCSF cells in 100 μL of phosphate-buffered saline (PBS) s.c. into their left flanks. Treatments were initiated after the tumors had reached about 50 mm^3^ in size. The first set of experiments investigated the correlation between the numbers of MDSCs and CSCs. Mice bearing ME180-control-derived or ME180-GCSF-derived tumors were intraperitoneally injected with anti-Gr-1-neutralizing antibody or PBS once a week for 4 weeks (*n* = 5). The second set of experiments examined the effect of PGE2 inhibition on CSCs. Mice bearing ME180-GCSF-derived tumors were intravenously treated with 5 mg/kg of daily celecoxib for 4 weeks (*n* = 5). The third set of experiments was conducted to investigate the antitumor effects of combination treatment involving celecoxib and cisplatin. Mice bearing ME180-control or ME180-GCSF-derived tumors were intraperitoneally injected with 4 mg/kg of weekly cisplatin, or with cisplatin plus celecoxib. Caliper measurements of the longest perpendicular diameter of each tumor were obtained once a week and used to estimate tumor volume according to the following formula: V = L × W × D × π/6, where V is the volume, L is the length, W is the width, and D is the depth. Furthermore, the serum PGE2 concentrations were evaluated using an enzyme-linked immunosorbent assay (ELISA).

### Isolation of MDSCs

MDSCs were isolated from the splenocytes of Balb/c mice using the Myeloid-Derived Suppressor Cell Isolation Kit and the MS column (Miltenyi Biotec, Auburn, CA, USA). The purity of the isolated cell population was previously determined using flow cytometry, and the frequency of CD11b^+^ Gr-1^+^ cells was >99% [16].

### Aldefluor assay

The Aldefluor Assay Kit (Stem Cell Technologies, Vancouver, Canada) was used to determine the percentage of tumor cells expressing high levels of ALDH (ALDH-high cells), according to the manufacturer’s instructions. Briefly, 1 × 10^6^ cells were incubated with the Aldefluor substrate for 45 minutes at 37° C, with and without the ALDH inhibitor, diethylaminobenzaldehyde (DEAB). After incubation, ALDH-high cells were detected in the FITC channel on a flow cytometer using the FACSDiva software.

### Sphere formation assay

ME180 cells were plated in ultra-low attachment surface 6-well plate with serum-free medium supplemented with bFGF (10 ng/ml; ReproCELL, Inc., Kanagawa, Japan), EGF (20 ng/ml; R&D Systems), and B27 supplement. After two weeks, the number of spheres in each well was counted using a phase-contrast microscope.

### Flow cytometry

Single-cell suspensions were prepared from mouse spleens and tumor specimens. Red blood cells were removed using ammonium chloride lysis buffer. Then, the cells were filtered through 40-μm nylon strainers, incubated with antibodies, and analyzed by flow cytometry. Flow cytometric data were acquired on a FACScan flow cytometer and analyzed using the FACSDiva software (BD Biosciences, San Jose, CA, USA). Cells that had been incubated with irrelevant isotype-matched antibodies and unstained cells served as controls.

### T cell proliferation assay

A 96-well plate was coated with 1 μg/well anti-CD3e antibody (Tonbo Biosciences, San Diego, CA, USA). CD8^+^ T cells were purified from human peripheral blood, using the CD8^+^ T Cell Isolation Kit and the MS column (Miltenyi Biotec, Auburn, CA, USA), according to the manufacturer’s instructions. MDSCs (CD11b^+^, CD33^+^, HLA-DR^-^) were sorted from tumors of uterine cervical cancer patients using flowcytometry. To determine the impact of MDSCs on T cell proliferation, MDSCs were co-cultured with CD8^+^ T cells. Cell proliferation was assayed using the cell proliferation ELISA BrdU kit (Roche Applied Science, Penzberg, Germany).

### Reverse transcriptase polymerase chain reaction (RT-PCR)

RNA was extracted from cells using TRIzol (Life Technologies, Grand Island, NY, USA). The resultant total RNA (1μg) was used to synthesize cDNA with ReverTraAce qPCR RT Master Mix (Toyobo, Osaka, Japan). The PCR was performed using Taq PCR master mix (Qiagen, Valencia, CA, USA) and specific primers. Amplification was conducted using a Takara PCR personal-type thermal cycler (Takara, Shiga, Japan). The PCR primers were purchased from Eurofins Genomics (Tokyo, Japan). The sequences of the primers used were as follows: β-actin: forward primer, 5′-CGTGACATTAAGGAGAAGCTGTG-3′ and reverse primer, 5′-GCTCAGGAGGAGCAATGATCTTGA-3′; EP2 receptor: forward primer, 5′-CAACCTCATCCGCATGCAC-3′ and reverse primer, 5′-CTCAAAGGTCAGCCTG-3′; EP4 receptor: forward primer, 5′-TGGTATGTGGGCTGGCTG-3′ and reverse primer, 5′-GAGGACGGTGGCGAGAAT-3′.

### Western blot analysis

The cells were lysed for 10 minutes at 4° C. Equal amounts of protein were separated by sodium dodecyl sulfate polyacrylamide gel electrophoresis and transferred to polyvinylidene difluoride membranes. Western blot analyses were conducted using various specific primary antibodies. The resultant immunoblots were visualized with horseradish peroxidase-coupled immunoglobulins using an enhanced chemiluminescence Western blotting system (PerkinElmer, Santa Clara, CA, USA). Signal intensities of COX-1 and COX-2 were quantified using ImageJ 1.52 g, and normalized to β actin. COX-1 antibody (#4841), COX-2 antibody (#4842), and β actin (#4967) antagonist and ONO-AE3-208 (an EP4 receptor antagonist) were obtained from Cell Signaling Technology (Beverly, MA, USA).

### Enzyme-linked immunosorbent assay (ELISA)

The PGE2 concentrations of media or serum were measured using the Prostaglandin E2 Express ELISA Kit (cat no. 500141) or the Prostaglandin E Metabolite ELISA Kit (cat no. 514531), which were both obtained from Cayman Chemical (Ann Arbor, MI, USA), respectively. Absorbance values were measured using a microplate reader (iMark™ Microplate Reader; Bio-Rad Laboratories, Inc., Hercules, CA, USA).

### Immunohistochemistry

Tumor samples were fixed in 10% neutral buffered formalin, embedded in paraffin, sectioned, and processed for immunohistochemical staining. The primary antibodies used were an anti-human CD33 monoclonal antibody (Clone, NCL-L-CD33, 1:100, Novocastra; Leica Biosystems, Wetzlar, Germany) and an anti-human ALDH1 monoclonal antibody (clone, 44/ALDH, 1:200, BD biosciences, San Jose, CA, USA). The slides were examined using a bright field microscope. The number of CD33^+^ cells was counted using a bright field microscope in low-power fields. The ALDH1 immunoreactivity in tumor cells was assessed using an immunoreactive score according to Remmele and Stegner (IRS). Optical images were captured using the PROVIS AX80 (Olympus, Tokyo, Japan).

### Statistical analysis

Continuous data were compared between the groups using the Student’s *t* test or the Wilcoxon rank sum test. *P*-values of < 0.05 were considered significant.

## SUPPLEMENTARY MATERIALS FIGURES



## Abbreviations

MDSCs: myeloid-derived suppressor cells
CSC: cancer stem-like cell
TAM: tumor-associated macrophage
CAFs: cancer-associated fibroblasts
PGE2: prostaglandin E2
G-CSF: granulocyte-colony stimulating factor
COX: cyclooxygenase
ALDH: aldehyde dehydrogenase
CtBP2: c-terminal binding protein-2
STAT3: signal transducer and activator of transcription 3
NO: nitric oxide
TRL: tumor-related leukocytosis

## References

[1] Torre LA, Bray F, Siegel RL, Ferlay J, Lortet-Tieulent J, Jemal A (2015). Global cancer statistics, 2012. CA Cancer J Clin.

[2] Eifel PJ, Winter K, Morris M, Levenback C, Grigsby PW, Cooper J, Rotman M, Gershenson D, Mutch DG (2004). Pelvic irradiation with concurrent chemotherapy versus pelvic and para-aortic irradiation for high-risk cervical cancer: an update of radiation therapy oncology group trial (RTOG) 90-01. J Clin Oncol.

[3] Dunn GP, Bruce AT, Ikeda H, Old LJ, Schreiber RD (2002). Cancer immunoediting: from immunosurveillance to tumor escape. Nat Immunol.

[4] Marvel D, Gabrilovich DI (2015). Myeloid-derived suppressor cells in the tumor microenvironment: expect the unexpected. J Clin Invest.

[5] Bonnet D, Dick JE (1997). Human acute myeloid leukemia is organized as a hierarchy that originates from a primitive hematopoietic cell. Nat Med.

[6] Pattabiraman DR, Weinberg RA (2014). Tackling the cancer stem cells - what challenges do they pose?. Nat Rev Drug Discov.

[7] López J, Poitevin A, Mendoza-Martínez V, Pérez-Plasencia C, García-Carrancá A (2012). Cancer-initiating cells derived from established cervical cell lines exhibit stem-cell markers and increased radioresistance. BMC Cancer.

[8] Kumazawa S, Kajiyama H, Umezu T, Mizuno M, Suzuki S, Yamamoto E, Mitsui H, Sekiya R, Shibata K, Kikkawa F (2014). Possible association between stem-like hallmark and radioresistance in human cervical carcinoma cells. J Obstet Gynaecol Res.

[9] Kim BW, Cho H, Choi CH, Ylaya K, Chung JY, Kim JH, Hewitt SM (2015). Clinical significance of OCT4 and SOX2 protein expression in cervical cancer. BMC Cancer.

[10] Chhabra R (2015). Cervical cancer stem cells: opportunities and challenges. J Cancer Res Clin Oncol.

[11] Chen WJ, Ho CC, Chang YL, Chen HY, Lin CA, Ling TY, Yu SL, Yuan SS, Chen YJ, Lin CY, Pan SH, Chou HY, Chen YJ (2014). Cancer-associated fibroblasts regulate the plasticity of lung cancer stemness via paracrine signalling. Nat Commun.

[12] Cui TX, Kryczek I, Zhao L, Zhao E, Kuick R, Roh MH, Vatan L, Szeliga W, Mao Y, Thomas DG, Kotarski J, Tarkowski R, Wicha M (2013). Myeloid-derived suppressor cells enhance stemness of cancer cells by inducing microRNA101 and suppressing the corepressor CtBP2. Immunity.

[13] Panni RZ, Sanford DE, Belt BA, Mitchem JB, Worley LA, Goetz BD, Mukherjee P, Wang-Gillam A, Link DC, Denardo DG, Goedegebuure SP, Linehan DC (2014). Tumor-induced STAT3 activation in monocytic myeloid-derived suppressor cells enhances stemness and mesenchymal properties in human pancreatic cancer. Cancer Immunol Immunother.

[14] Peng D, Tanikawa T, Li W, Zhao L, Vatan L, Szeliga W, Wan S, Wei S, Wang Y, Liu Y, Staroslawska E, Szubstarski F, Rolinski J (2016). Myeloid-Derived Suppressor Cells Endow Stem-like Qualities to Breast Cancer Cells through IL6/STAT3 and NO/NOTCH Cross-talk Signaling. Cancer Res.

[15] Wang D, Fu L, Sun H, Guo L, DuBois RN (2015). Prostaglandin E2 Promotes Colorectal Cancer Stem Cell Expansion and Metastasis in Mice. Gastroenterology.

[16] Mabuchi S, Matsumoto Y, Kawano M, Minami K, Seo Y, Sasano T, Takahashi R, Kuroda H, Hisamatsu T, Kakigano A, Hayashi M, Sawada K, Hamasaki T (2014). Uterine cervical cancer displaying tumor-related leukocytosis: a distinct clinical entity with radioresistant feature. J Natl Cancer Inst.

[17] Kawano M, Mabuchi S, Matsumoto Y, Sasano T, Takahashi R, Kuroda H, Kozasa K, Hashimoto K, Isobe A, Sawada K, Hamasaki T, Morii E, Kimura T (2015). The significance of G-CSF expression and myeloid-derived suppressor cells in the chemoresistance of uterine cervical cancer. Sci Rep.

[18] Mao Y, Sarhan D, Steven A, Seliger B, Kiessling R, Lundqvist A (2014). Inhibition of tumor-derived prostaglandin-e2 blocks the induction of myeloid-derived suppressor cells and recovers natural killer cell activity. Clin Cancer Res.

[19] Kalinski P (2012). Regulation of immune responses by prostaglandin E2. J Immunol.

[20] Millrud CR, Bergenfelz C, Leandersson K (2017). On the origin of myeloid-derived suppressor cells. Oncotarget.

[21] Liu SY, Zheng PS (2014). High aldehyde dehydrogenase activity identifies cancer stem cells in human cervical cancer. Oncotarget.

[22] Wang L, Guo H, Lin C, Yang L, Wang X (2014). Enrichment and characterization of cancer stem-like cells from a cervical cancer cell line. Mol Med Rep.

[23] Bortolomai I, Canevari S, Facetti I, De Cecco L, Castellano G, Zacchetti A, Alison MR, Miotti S (2010). Tumor initiating cells: development and critical characterization of a model derived from the A431 carcinoma cell line forming spheres in suspension. Cell Cycle.

[24] Wang XY, Yi H, Li J (2016). Response to: ‘Issues with anti-Gr1 antibody-mediated myeloid-derived suppressor cell depletion’ by Xing et al. Ann Rheum Dis.

[25] Sasano T, Mabuchi S, Kozasa K, Kuroda H, Kawano M, Takahashi R, Komura N, Yokoi E, Matsumoto Y, Hashimoto K, Sawada K, Morii E, Kimura T (2018). The Highly Metastatic Nature of Uterine Cervical/Endometrial Cancer Displaying Tumor-Related Leukocytosis: Clinical and Preclinical Investigations. Clin Cancer Res.

[26] Kurtova AV, Xiao J, Mo Q, Pazhanisamy S, Krasnow R, Lerner SP, Chen F, Roh TT, Lay E, Ho PL, Chan KS (2015). Blocking PGE2-induced tumour repopulation abrogates bladder cancer chemoresistance. Nature.

[27] Zarghi A, Arfaei S (2011). Selective COX-2 Inhibitors: A Review of Their Structure-Activity Relationships. Iran J Pharm Res.

[28] Veltman JD, Lambers ME, van Nimwegen M, Hendriks RW, Hoogsteden HC, Aerts JG, Hegmans JP (2010). COX-2 inhibition improves immunotherapy and is associated with decreased numbers of myeloid-derived suppressor cells in mesothelioma. Celecoxib influences MDSC function. BMC Cancer.

[29] Fujita M, Kohanbash G, Fellows-Mayle W, Hamilton RL, Komohara Y, Decker SA, Ohlfest JR, Okada H (2011). COX-2 blockade suppresses gliomagenesis by inhibiting myeloid-derived suppressor cells. Cancer Res.

[30] Derry S, Moore RA (2013). Single dose oral celecoxib for acute postoperative pain in adults. Cochrane Database Syst Rev.

[31] Farley JH, Truong V, Goo E, Uyehara C, Belnap C, Larsen WI (2006). A randomized double-blind placebo-controlled phase II trial of the cyclooxygenase-2 inhibitor Celecoxib in the treatment of cervical dysplasia. Gynecol Oncol.

[32] Kryczek I, Liu S, Roh M, Vatan L, Szeliga W, Wei S, Banerjee M, Mao Y, Kotarski J, Wicha MS, Liu R, Zou W (2012). Expression of aldehyde dehydrogenase and CD133 defines ovarian cancer stem cells. Int J Cancer.

[33] Wicha MS (2006). Cancer stem cells and metastasis: lethal seeds. Clin Cancer Res.

[34] Batlle E, Clevers H (2017). Cancer stem cells revisited. Nat Med.

[35] Taki M, Abiko K, Baba T, Hamanishi J, Yamaguchi K, Murakami R, Yamanoi K, Horikawa N, Hosoe Y, Nakamura E, Sugiyama A, Mandai M, Konishi I (2018). Snail promotes ovarian cancer progression by recruiting myeloid-derived suppressor cells via CXCR2 ligand upregulation. Nat Commun.

[36] Bruhn KW, Dekitani K, Nielsen TB, Pantapalangkoor P, Spellberg B (2015). Ly6G-mediated depletion of neutrophils is dependent on macrophages. Results Immunol.

[37] Yoshida Y, Sadata A, Zhang W, Saito K, Shinoura N, Hamada H (1998). Generation of fiber-mutant recombinant adenoviruses for gene therapy of malignant glioma. Hum Gene Ther.

[38] Samulski RJ, Srivastava A, Berns KI, Muzyczka N (1983). Rescue of adeno-associated virus from recombinant plasmids: gene correction within the terminal repeats of AAV. Cell.

