# Supplementary material for: Prostaglandin E2 produced by myeloid-derived suppressive cells induces cancer stem cells in uterine cervical cancer
Source: Oncotarget. 2018 Nov 20;9(91):36317–30. doi: 10.18632/oncotarget.26347 (PMC6284736; doi:10.18632/oncotarget.26347)
Supplement: Supplementary file 1 [file oncotarget-09-36317-s001.pdf]

Prostaglandin E2 produced by myeloid-derived suppressive cells induces cancer stem cells in uterine cervical cancer

SUPPLEMENTARY MATERIALS

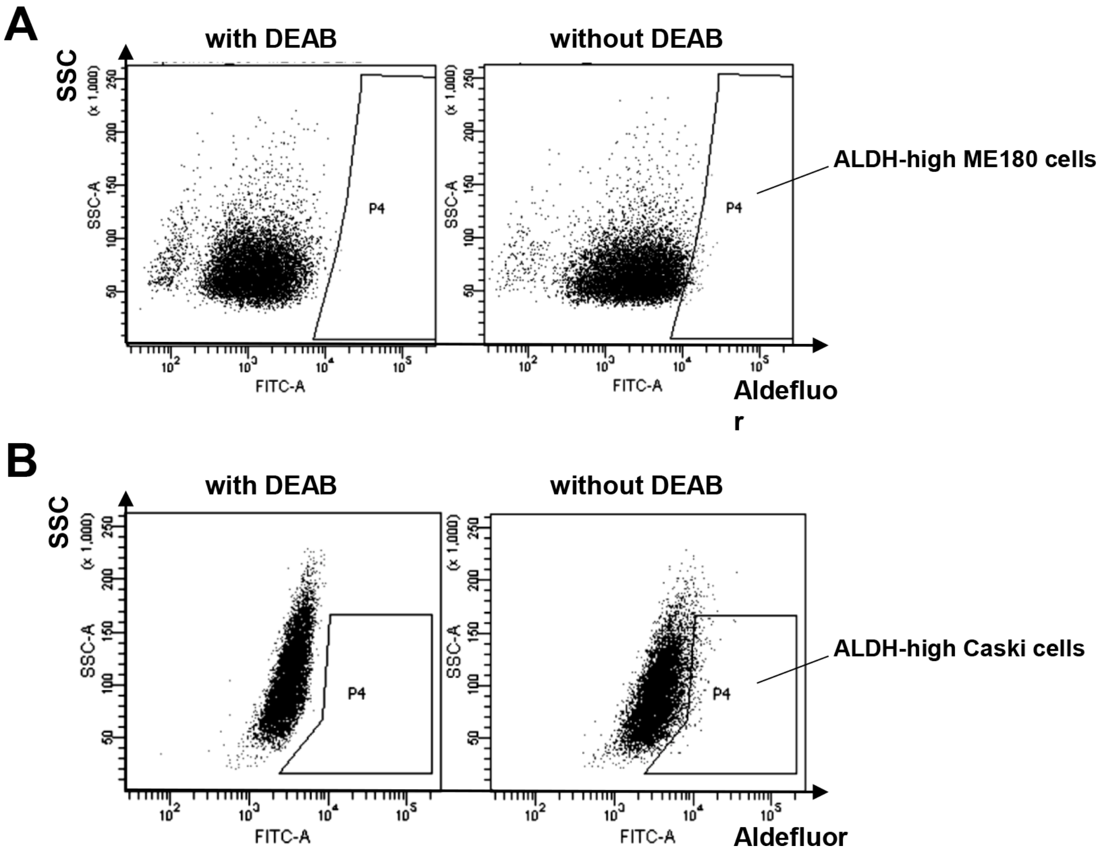

Supplementary Figure 1: ALDH activity of ME180 cells (**A**) and CaSki cells (**B**) *in vitro*. 3×10^6 cells of ME180 cells and CaSki cells were cultured for 3 days in DMEM supplemented with 10% FBS, and their ALDH activity were assessed using the Aldefluor assay. The representative dot plots of Aldefluor assay with DEAB (an ALDH-inhibitor) are shown in the left side, and those with DEAB on the right side.

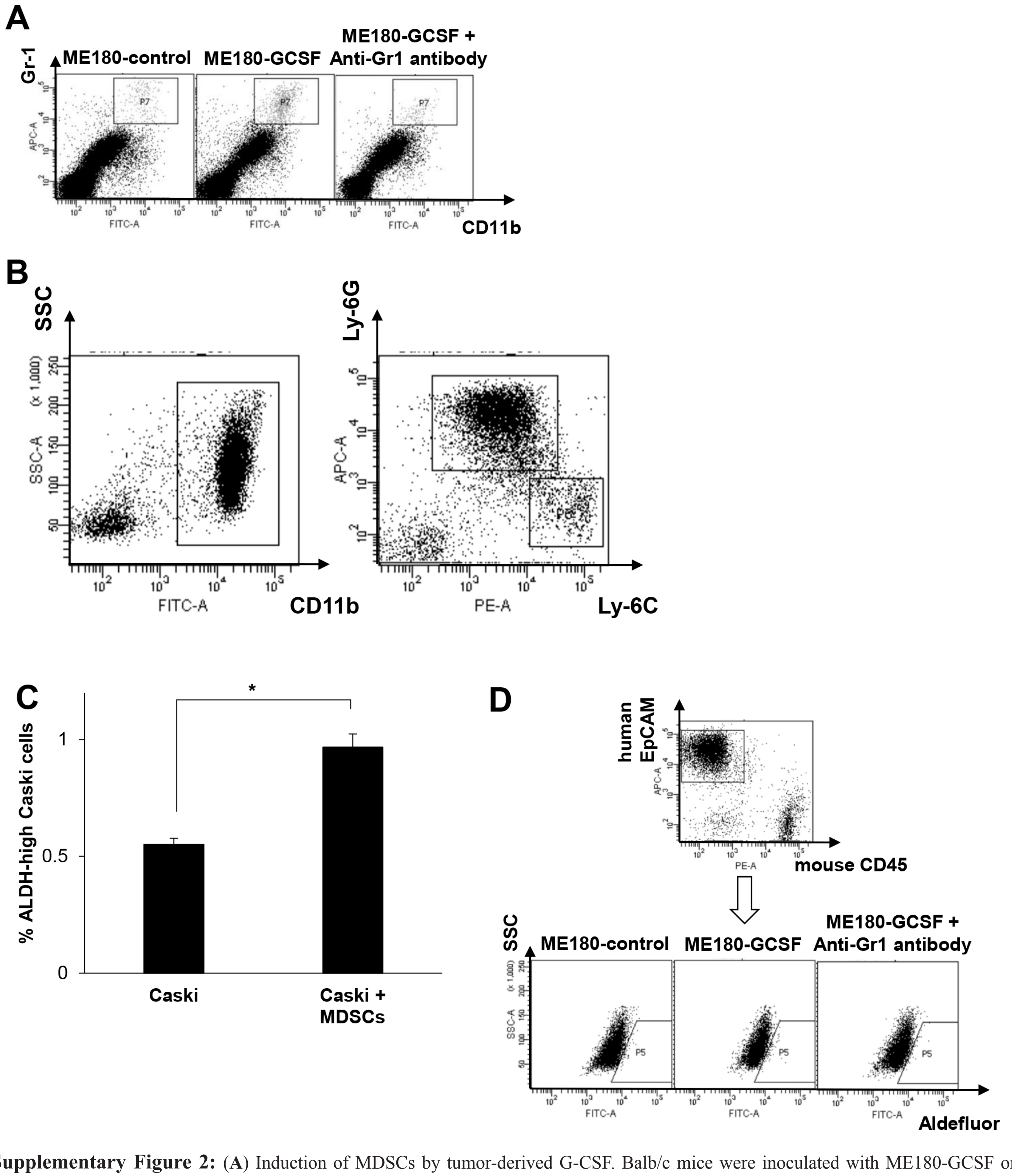

Supplementary Figure 2: (**A**) Induction of MDSCs by tumor-derived G-CSF. Balb/c mice were inoculated with ME180-GCSF or ME180-control cells. Four weeks after the inoculation, their subcutaneous tumors were collected and assessed for MDSCs using flow cytometry for MDSCs. Representative dot plots are shown. (**B**) Granulocytic and monocytic MDSCs subsets. $CD11b^{+}Gr1^{+}$ cells from the spleens of mice bearing ME180-GCSF-derived tumors were collected by magnetic bead selection. The $CD11b^{+}$ cells were gated and then re-plotted for their Ly6G and Ly6C expression to determine the frequencies of the granulocytic and monocytic MDSCs subsets. More than 80% of the isolated MDSCs co-expressed CD11b and Gr-1, and almost 83% of them were positive for $Ly6G^{+}$. (**C**) Effect of MDSCs on the induction of CSCs *in vitro*. CaSki cells were cultured with or without MDSCs in the presence of 0.1% of FBS for 12 hours (5:1 ratio of ME180: MDSCs). The frequencies of ALDH-high ME180 cells were assessed using the Aldefluor assay. Splenocytes excluding MDSCs were used as a negative control. (Bars SD. $n = 6$, $p < 0.01$, two-sided Student's t test). (**D**) The frequency of CSCs in an *in vivo* cervical cancer model. Balb/c mice were inoculated with ME180-GCSF or ME180-control cells. Four weeks after the inoculation, their subcutaneous tumors were collected. The human $EpCam^{+}$ mouse $CD45^{-}$ cells in the tumors were gated using flow cytometry and then the percentages of ALDH-high cells were assessed using the Aldefluor assay. Representative dot plots are shown.

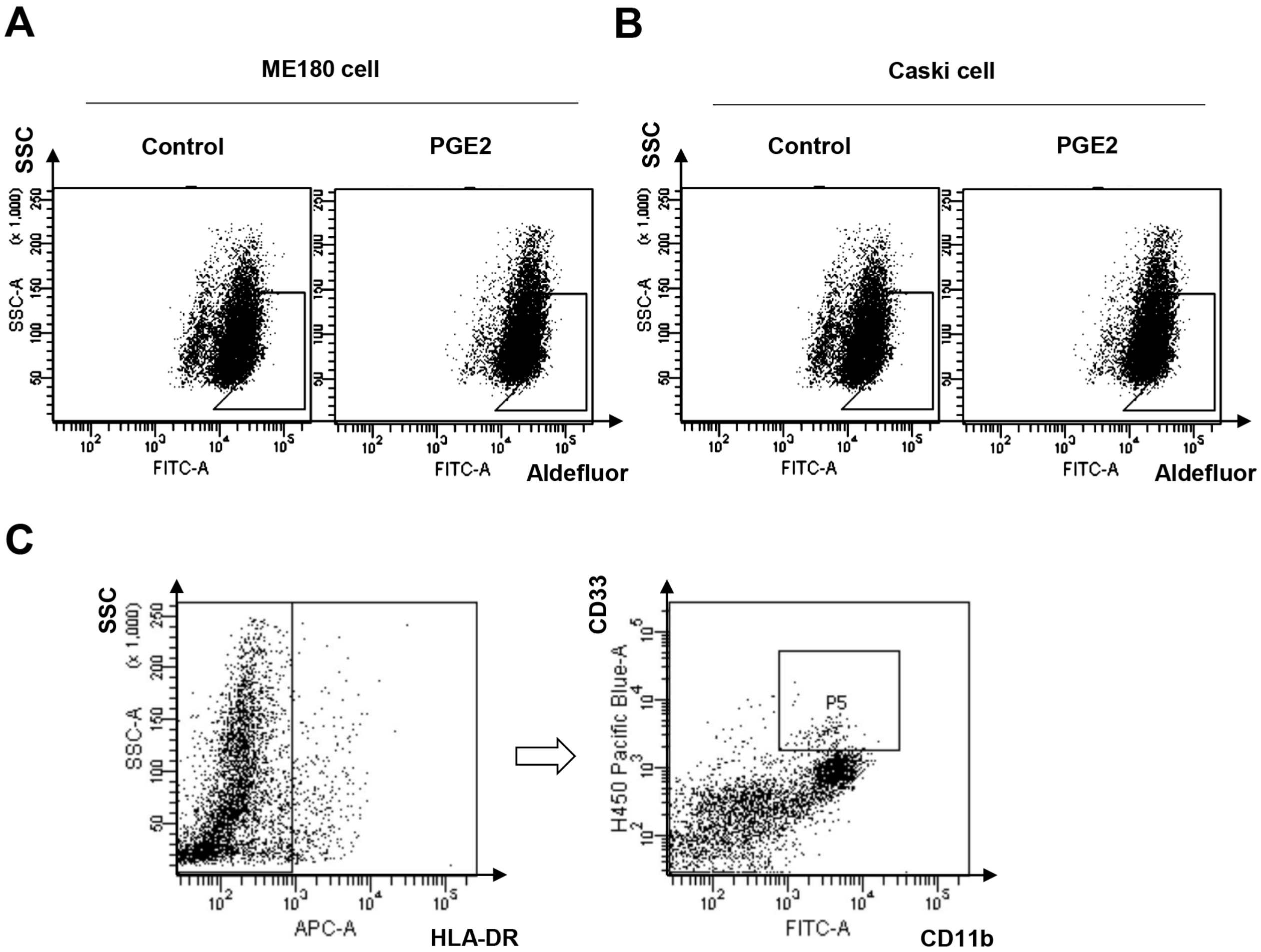

Supplementary Figure 3: (**A**, **B**) Effect of PGE2 on the induction of CSCs *in vitro*. ME180 (A) or CaSki (B) cervical cancer cells were cultured for 18 hours in the presence or absence of 0.1 μM of PGE2 *in vitro*. Then, the frequencies of ALDH-high ME180 cells were assessed using the Aldefluor assay. Representative dot plots are shown. (**C**) Human MDSCs subset. The percentages of CD11b$^+$CD33$^+$HLA-DR$^-$ cells in cervical cancers were assessed using flow cytometry. Representative dot plots of Figure 5B are shown.

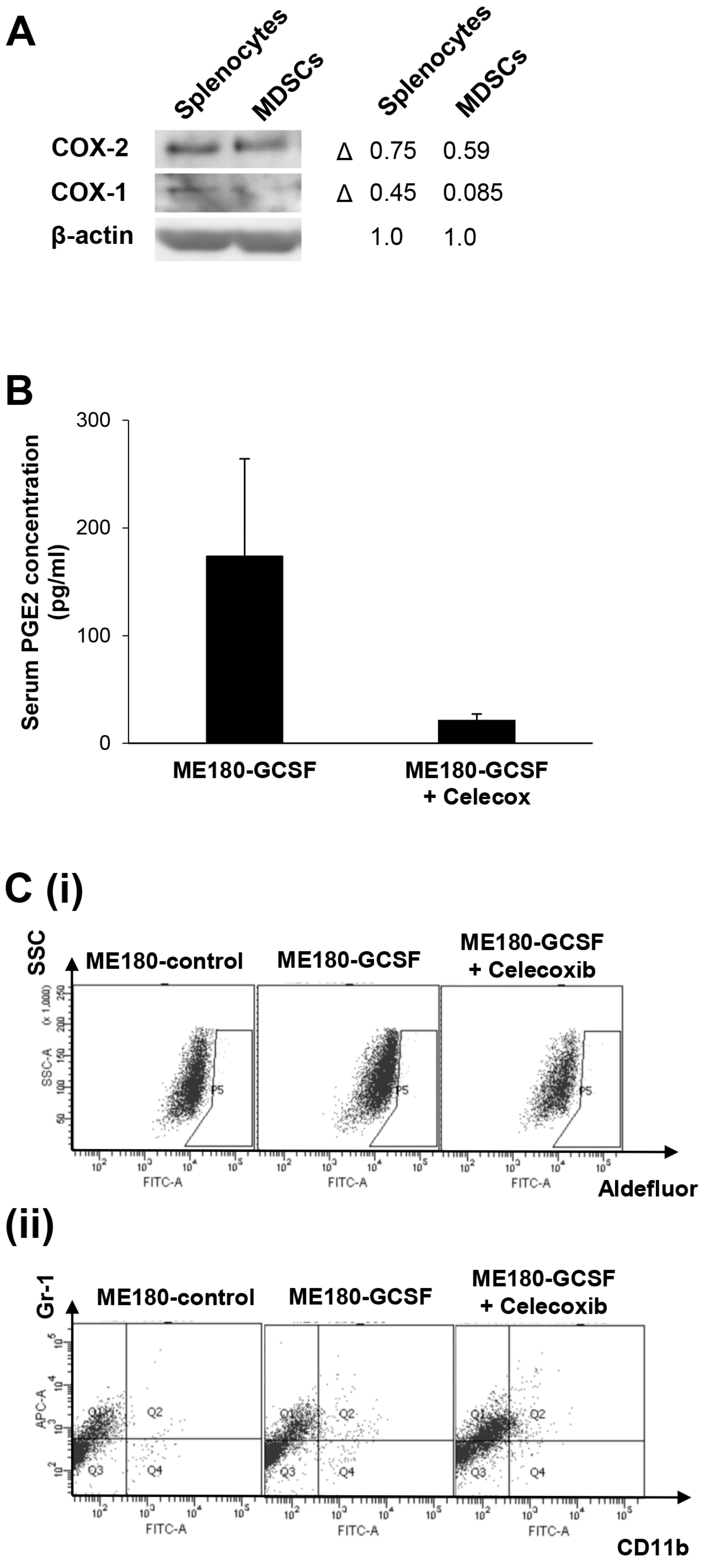
A
Splenocytes
MDSCs
Splenocytes
MDSCs
COX-2
Δ 0.75 0.59
COX-1
Δ 0.45 0.085
β-actin
1.0 1.0
B
Serum PGE2 concentration (pg/ml)
300
200
100
0
ME180-GCSF
ME180-GCSF + Celecox
C (i)
SSC
ME180-control
ME180-GCSF
ME180-GCSF + Celecoxib
Aldefluor
(ii)
Gr-1
ME180-control
ME180-GCSF
ME180-GCSF + Celecoxib
CD11b

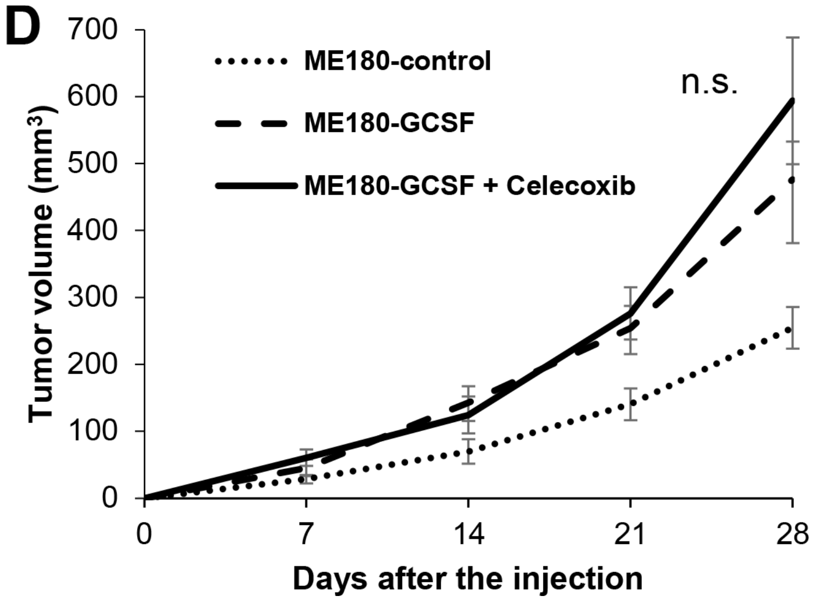

Supplementary Figure 4: (**A**) The expression of COX-1 and COX-2 in mouse MDSCs. The expression of COX-1 and COX-2 in mouse MDSCs, compared to splenocytes of ME180-GCF-derived tumor bearing mice (Western blotting). Δ, fold change by densitometry (fold change normalized to β-actin) (**B**). Effect of celecoxib on the serum PGE2 concentration. The serum PGE2 concentrations of the tumor-bearing mice 4 weeks after the inoculation were assessed using the Prostaglandin E Metabolite ELISA Kit (Bars SD. $n = 5$, $p < 0.01$, two-sided Student's *t* test). (**C**) *In vivo* effect of PGE2-inhibition on the induction of CSCs. Balb/c mice were inoculated with ME180-GCSF or ME180-control cells. 2 weeks after the inoculation, mice bearing ME180-GCSF-derived tumors were randomly assigned to 2 treatment groups: 5 mg/kg of daily celecoxib (i.p.) or PBS. C (i). Effect of celecoxib on the induction of CSCs in tumors. Representative dot plots of Figure 5A are shown. C (ii). Effect of celecoxib on the induction of MDSCs in tumors. Representative dot plots of Figure 5B are shown. (**D**) Effect of the celecoxib in a mouse cervical cancer model. Balb/c nude mice were inoculated with ME180-GCSF or ME180-control cells. Two weeks after the inoculation, the ME180-GCSF mice were assigned to 2 groups: PBS ($n = 5$), 5 mg/kg of daily celecoxib ($n = 5$). The volumes of the tumors were measured for 4 weeks after the inoculation (Bars SD. $n = 5$, $p < 0.05$, two-sided Student's *t* test).
